# The Impact of Obesity and Associated Comorbidities on the Outcomes after Renal Transplantation with a Living Donor vs. Deceased Donor Grafts

**DOI:** 10.3390/jcm11113069

**Published:** 2022-05-29

**Authors:** Renana Yemini, Ruth Rahamimov, Eviatar Nesher, Roi Anteby, Ronen Ghinea, Tammy Hod, Eytan Mor

**Affiliations:** 1 Department of Surgery, Samson Assuta Ashdod University Hospital, Ashdod 7747629, Israel; 2 Faculty of Health Sciences, Ben Gurion University of the Negev, Beer-Sheva 8410501, Israel; 3 Institute of Nephrology, Beilinson Medical Center, Petach-Tikva 49100, Israel; rutir@clalit.org.il; 4 Sackler Medical School, Tel-Aviv University, Tel Aviv 6997801, Israel; eviatarne@clalit.org.il (E.N.); anteby.roi@gmail.com (R.A.); ronen.ghinea@sheba.health.gov.il (R.G.); tamar.hod@sheba.health.gov.il (T.H.); eytan.mor@sheba.health.gov.il (E.M.); 5 Department of Transplant Surgery, Beilinson Medical Center, Petach-Tikva 49100, Israel; 6 Transplant Center, Department of Surgery B, Sheba Medical Center, Ramat Gan 5266202, Israel; 7 Transplant Center, Department of Nephrology, Sheba Medical Center, Ramat Gan 5266202, Israel

**Keywords:** kidney transplant, obesity, living and deceased related kidneys grafts, graft rejection

## Abstract

Background: Obesity among kidney transplant (KT) recipients can lead to metabolic comorbidity-associated deaths. This study compares post-KT survival between obese and non-obese patients and outcomes of living donor (LD) and deceased donor (DD) grafts. Methods: Between January 2005–May 2019, 1403 KT recipients from a single center were included in the study, as well as 314 patients (22.4%) with obesity (BMI > 30 kg/m^2^), 137 DD transplants, and 177 LD transplants. Of the 1089 (77.6%) in the control group (BMI ≤ 30 kg/m^2^), 384 were DD transplants and 705 LD transplants. The Kaplan–Meier method was used for survival analysis and a Cox regression was used to identify risk factors for graft loss and mortality. Propensity score matching analysis adjusting for age, IHD, and T2DM was performed. Results: The study group had higher incidence of obesity related comorbidities, delayed graft function and primary non function (*p* < 0.001). One-, 5-and 10-year patient and graft survival were lower in the study group (*p* < 0.001). Subgroup analysis of graft survival according to type of graft shows a difference in the DD (*p* = 0.002) but not in the LD group (*p* = 0.220). However, mortality was higher in both groups (LD, *p* = 0.045; DD, *p* = 0.004). Risk factors for mortality were age, T2DM, IHD, and DD, and for graft failure: IHD, BMI, donor age, re-transplant, and DD. Propensity score analysis shows an odds ratio of 0.81 for graft failure and 0.93 for death in the study group (95% CI = 0.55, 1.21, *p* = 0.3 and CI = 0.59, 1.46, *p* = 0.7, respectively). Conclusions: Recipient age and metabolic comorbidities should be emphasized when evaluating patients with obesity. We suggest considering weight loss interventions using the new GLP-1 inhibitors and bariatric procedures in selected patients to prepare overweight patients for transplant.

## 1. Introduction

Obesity has reached pandemic proportions and continues to be a growing problem worldwide, with more than one-third of the population meeting the criteria for frank obesity [1,2]. It is commonly associated with metabolic syndrome, inducing and exacerbating comorbidities, such as heart disease, renal disease, and nonalcoholic steatohepatitis, which, in turn, can lead to end-stage organ disease [3,4]. A special population among obese patients is a subgroup of those who undergo organ transplantation. The high prevalence of obesity among those patients is mainly due to the fact that obesity promotes end-stage organ disease, and that transplantation causes an increase in body weight [5,6]. A body mass index (BMI) > 40 kg/m^2^ is a contraindication for listing in many transplant programs, and some will not accept patients with a BMI > 35 kg/m^2^ until they lose weight. Furthermore, obesity is associated with a high risk of surgical complications immediately after transplant [7,8], as well as higher mortality rates and graft failure in the long-term [9]. A recent study by Scheuermann et al. showed that obese KT recipients were significantly more likely to experience surgical complications such as wound infections, fascial dehiscence, and lymphoceles. In addition, recipient obesity was found to be an independent risk factor of delayed graft function (DGF) [10].

Based on a 20-year follow-up, Hoogeveen reported that the one-year post-transplant BMI and the BMI increment are more substantially related to death and graft failure than the pre-transplant BMI among kidney transplant recipients [11]. They showed that patients with a BMI > 30 kg/m^2^ have an approximately 20–40% higher risk of death and graft failure compared to patients with a normal BMI. Meier-Kriesche et al.’s estimation of the impact of various degrees of obesity revealed that patients with morbid obesity (MO) (BMI > 35 kg/m^2^) had lower patient and graft survival rates at 1 and 5 years compared to the moderately obese group (BMI 30–34.9 kg/m^2^) [9,12].

There is currently a lack of data on how the type of kidney graft donation, i.e., from living (LD) and deceased donors (DD), affects the results of transplantation in this large subgroup of obese transplanted patients. The present study aimed to retrospectively investigate the effect of obesity on post-transplant graft and patient survival among recipients of a kidney from a living donor (LD) compared to a kidney from a deceased donor (DD). We anticipated that the study results will provide a better insight into the problem of obesity in the context of kidney transplantation and help in forming recommendations for surgical decision-making as well as in preparing this exceptionally high-risk group of patients for transplantation.

## 2. Methods

### 2.1. Patient Selection

The prospective data collection and retrospective review were approved by the Institutional Review Board of our Center. All kidney transplant patients who underwent transplantation in our institute were studied prospectively between January 2005 to May 2019. There were a total 1403 transplanted patients available for analysis after excluding patients with missing anthropometric data, patients who underwent co-transplantation of another organ (heart, liver, or pancreas), and patients who had undergone bariatric surgery. In a retrospective analysis, we compared the outcome among two groups of patients who received kidney grafts from either an LD or a DD. The study group was comprised of patients with obesity (BMI > 30 kg/m^2^) and compared to the control group, which composed of patients with normal weight to overweight (BMI 18–30 kg/m^2^). The patients were referred by their nephrologist to the transplantation clinic, and they were all approved for kidney transplantation by a multi-disciplinary team. There had been no BMI cutoff to accept patients for transplants until 2012, after which a cutoff of 35 kg/m^2^ was instituted with a recommendation to lose weight by dietary measures or bariatric surgery (BS) prior to transplant.

Data were extracted from the medical records of the relevant hospital departments, including outpatient clinics, surgery, and anesthesia, and they consisted of the recipient’s and donor’s age and sex, cause of end-stage renal disease (ESRD; diabetic nephropathy, hypertensive disease, polycystic kidneys disease, focal and segmental glomerulosclerosis, glomerulonephritis, pyelonephritis, congenital, others, and unknown), preoperative weight and BMI (kg/m^2^), comorbidities (type 2 diabetes [T2DM] and ischemic heart disease [IHD]), dialysis duration before transplantation, graft from an LD or DD, panel reactive antibody (PRA), and HLA-DR mismatch (MM). Outcomes were determined by analysis of the patients’ records with a 10-year post-operation follow-up.

### 2.2. Perioperative Management

Maintenance immunosuppression included the calcineurin inhibitor tacrolimus (Prograf, Astellas Pharma, Middlesex, UK), starting on postoperative day 1 at a dose of 0.15 mg/kg, targeting 12-hour trough levels of 8 to 12 ng/mL during the first 3 months and 5 to 8 ng/mL thereafter, or cyclosporine (Sandimmune Neoral, Novartis Pharmaceutical) starting on postoperative day 1 at a dose of 8 mg/kg, targetin 12-hour trough levels of 150 to 300 ng/mL during the first 3 months and 100 to 200 ng/mL thereafter. Antiproliferative agents included mycophenolate mofetil (Cellcept, Roche Pharmaceuticals) at 1000 mg, twice daily for the first 2 weeks and 500 mg 3 times per day thereafter, or mycophenolic acid (Myfortic, Novartis Pharma) at 720 mg twice daily for the first 2 weeks and 360 mg 3 times per day thereafter. All patients received perioperative intravenous corticosteroid therapy with methylprednisolone at 500 mg on day 0, 250 mg on day 1, and 100 mg on day 2, after which they received oral prednisone of 20 mg per day, tapered to 5 mg per day within 3 months. Induction therapy consisted of one of the following: the anti–IL-2 receptor antagonist basiliximab (Simulect, Novartis Pharma) administered intravenously on days 0 and 4 at a dosage of 20 mg, daclizumab (Zenapax; Roche Pharmaceuticals, Basel, Switzerland) at a dosage of 1 mg/kg on days 0 and 14, or in cases of immunologic high risk, rabbit antithymocyte globulin (ATG) (Thymoglobulin; Genzyme Corp) at an intravenous dosage of 1.0–1.5 mg/kg daily for 3 days starting intraoperatively. Part of the study population did not receive induction therapy.

### 2.3. Clinical Outcomes

The primary clinical outcomes of this study were graft failure (defined as death or return to dialysis), death-censored graft failure, and all-cause mortality. Data on the incidence of any acute rejection were collected starting from January 2005. The reporting of acute rejection was coded according to Banff’s criteria and graded as mild, moderate, and severe. The outcome data of all recipients were censored on August 2019.

### 2.4. Statistical Analysis

Mean values, standard deviations, and absolute and relative frequencies were calculated for descriptive statistical analysis. Chi-squared tests were used to assess the difference in the frequencies between the two groups for categorical variables, and t-tests and analysis of variance (ANOVA) were applied for continuous variables. Variables that were significant on the univariable analysis were entered into a multivariable analysis. *p* values ≤ 0.05 were considered significant. Survival analysis was estimated using the Kaplan–Meier method, with the log-rank test for comparisons between groups and the Cox regression analysis applied for identifying risk factors for graft loss and demise. Results were expressed as a hazard ratio (HR) or as an odds ratio (OR) with a 95% confidence interval (CI). The covariates included in the logistic regression and Cox regression models were donor characteristics (age and gender), recipient characteristics (age, gender, cause of ESRD, pre-emptive transplantation, dialysis duration, T2DM, ischemic heart disease [IHD], weight, and BMI), and transplant-related characteristics. Effect modification between donor types with covariates and outcomes was also examined. Variables that had an association with clinical outcomes with *p*-values of <0.1 in the unadjusted analyses were included in the multivariable-adjusted analyses. A propensity score with a 1:1 optimal pair matching was conducted adjusting for age, IHD, and T2DM. The standardized mean differences threshold was set to 0.12. Statistical analyses were performed by IBM SPSS Statistics, software version 26.0 (IBM Corp., Armonk, NY) and by “MatchIt” and “cobalt” packages in R software (version 3.6.2, R Core Team, Vienna, Austria).

## 3. Results

**Figure 1:**
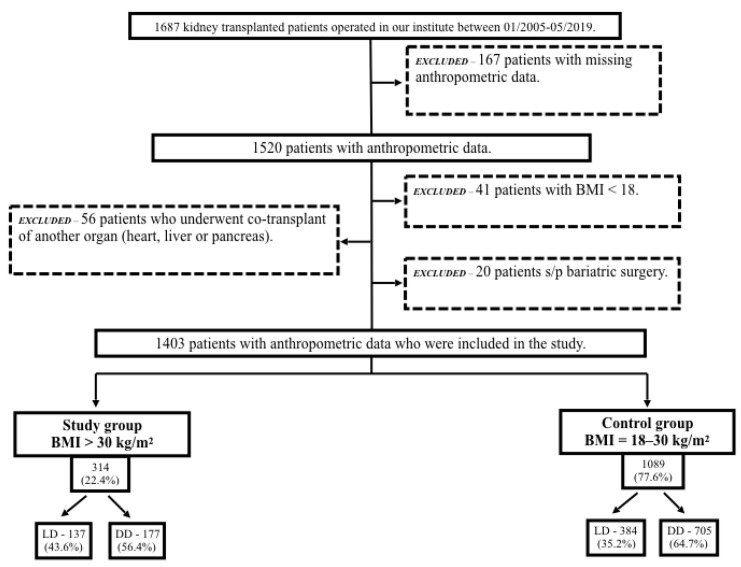
Patient selection diagram. BMI, body mass index; LD, living donor; DD, deceased donor; Tx, transplant.

A total of 1687 kidney transplants were performed in our institution between January 2005–August 2019. Excluded from this study were patients with missing anthropometric data (n = 167), those who underwent a co-transplant of another organ (heart, liver, or pancreas) (n = 56), those who had undergone bariatric surgery (n = 20), and those with BMIs < 18 kg/m^2^ (n = 41). Of the 1403 patients with anthropometric data who were included in the study, 314 (22.4%) were in the study group and 1089 (77.6%) were in the control group. Figure 1 displays the patient selection diagram and distribution of LD and DD grafts.

Demographic and characteristics of the patients, including age, gender, weight and BMI, comorbidities (T2DM and IHD), cause of ESRD, pre-emptive transplantation, dialysis duration, donor age, and type of donation, are presented in Table 1. There were significant differences in recipient age, comorbidities (T2DM and IHD) (*p* < 0.001), cause of ESRD, and pre-emptive transplantation between the groups (*p* = 0.037). Time on dialysis prior to transplant was significantly different between the DD and LD transplant subgroups (*p* < 0.001). The mean donor age of the control group was significantly younger compared to that of the study group (*p* = 0.005). Patients in the study group had a higher incidence of T2DM and diabetic nephropathy as the etiology of renal failure compared to patients in control group (*p* < 0.001). The rate of IHD was higher among patients with a higher BMI (study group; 33% vs. control 18.5%; *p* < 0.001). Among the study group, 56.4% of transplants were from living donor grafts, compared to 64.6% in the control group (*p* = 0.008).

Kidney transplant outcomes are presented in Table 2. There was a higher mean (± SD) length of stay after transplantation among the study group (14.28 ± 25.27 days), compared to the control group (10.89 ± 12.849 days) (*p* = 0.002). Furthermore, the incidence of delayed graft function (DGF( and primary non function (PNF) were significantly higher in patients with a BMI > 30 (*p* < 0.001).

**Figure 2:**
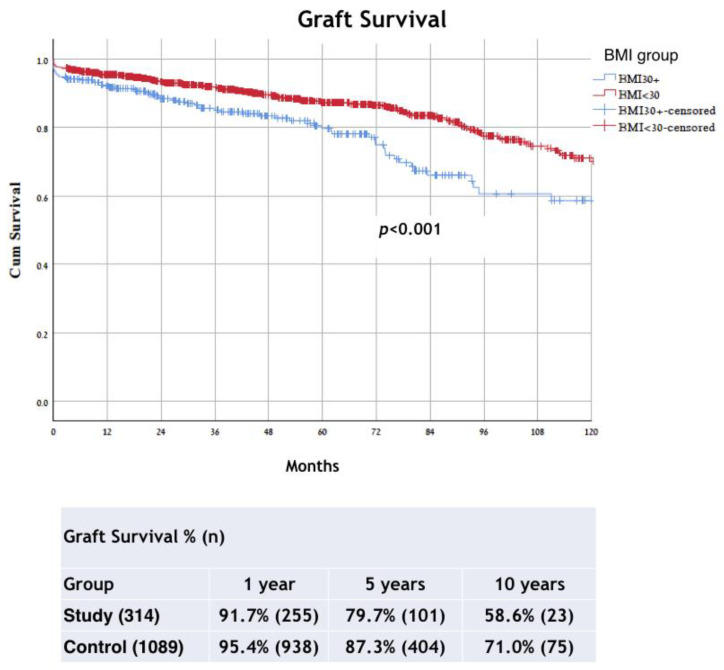
Kaplan–Meier method with log rank test for comparison graft survival analysis between groups. BMI, body mass index.

The respective 1-, 5-, and 10-year graft survival rates were 91.7%, 79.7%, and 58.6% for the study group and 95.4%, 87.3%, and 71.0% for the control group (*p* < 0.001) (Figure 2).

**Figure 3:**
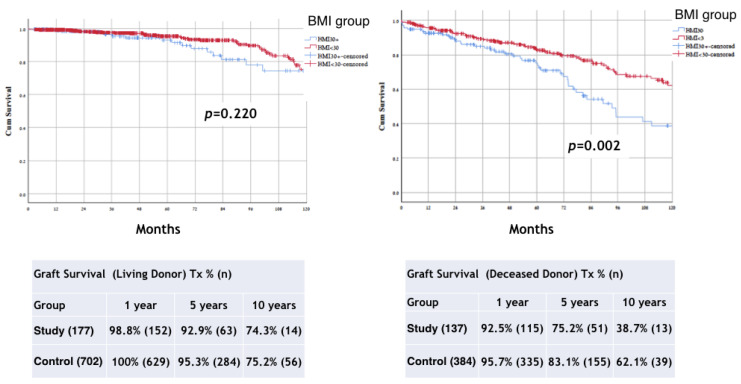
Kaplan–Meier method with log rank test for comparison graft survival (Living and Deceased Tx) analysis between groups. BMI, body mass index; Tx, transplant.

The subgroup analysis results in the BMI groups of graft survival according to type of graft shows a difference only in the DD group (*p* = 0.002), but not in the LD group (*p* = 0.220) (Figure 3).

**Figure 4:**
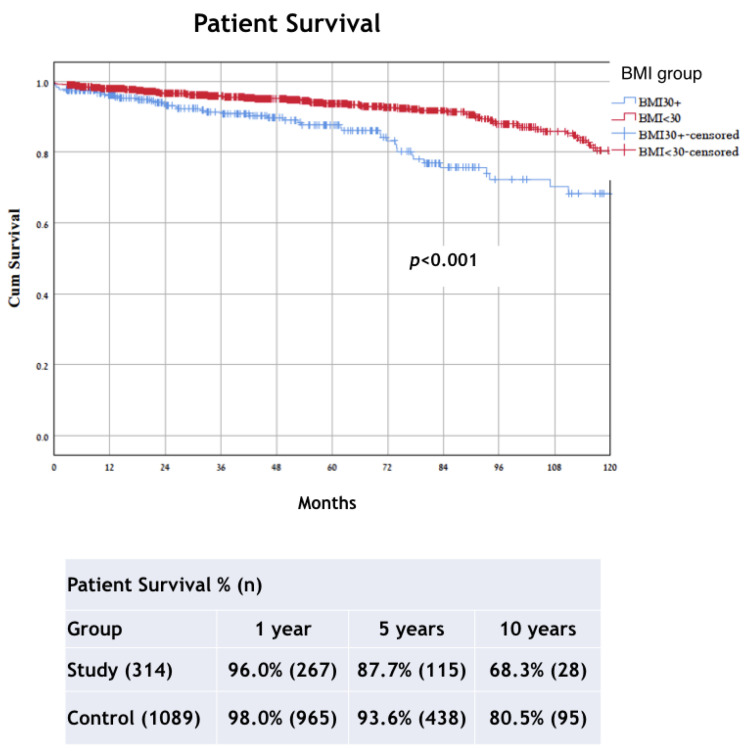
Kaplan–Meier method with log rank test for comparison patient survival analysis between groups. BMI, body mass index.

**Figure 5:**
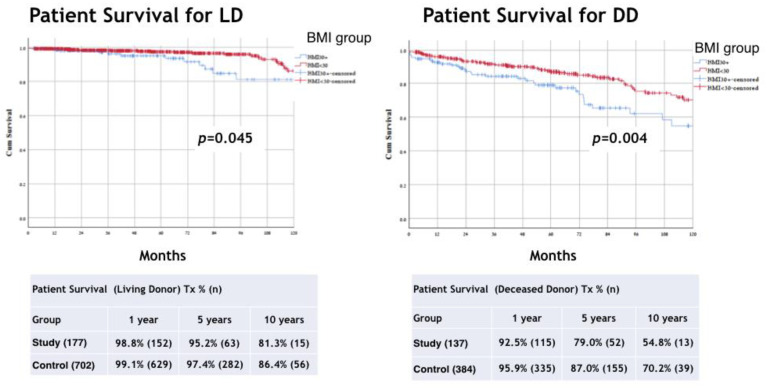
Kaplan–Meier method with log rank test for comparison patient survival (Living and Deceased Tx) analysis between groups. BMI, body mass index; Tx, transplant.

The respective 1-, 5-, and 10-year patient survival rates were 96.0%, 87.7%, and 68.3% for the study group and 98.0%, 93.6%, and 80.5% for the control group (*p* < 0.001) (Figure 4). The subgroup analysis results in the BMI groups of patient survival according to type of graft shows a difference in both LD and DD groups (LD, *p* = 0.045; DD, *p* = 0.004) (Figure 5).

**Figure 6:**
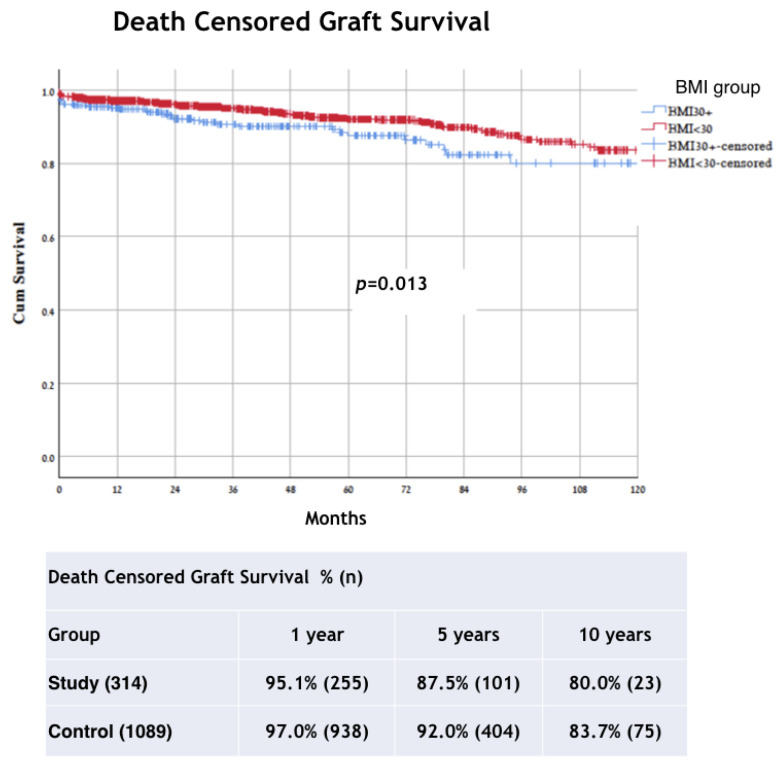
Kaplan–Meier method with log rank test for comparison death-censored graft survival analysis between groups. BMI, body mass index.

**Figure 7:**
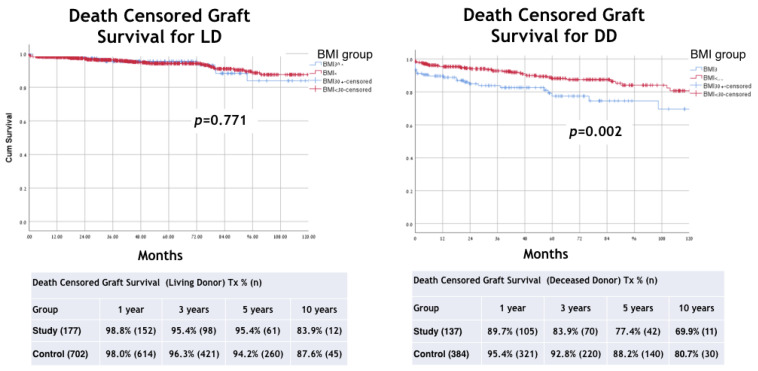
Kaplan-Meier method with log rank test for comparison death-censored graft survival (Living and Deceased Tx) analysis between groups. BMI, body mass index; Tx, transplant.

The respective 1-, 5-, and 10-year death-censored graft survival rates were 95.1%, 87.5%, and 80.0% in the study group and 97.0%, 92.0%, and 83.7% in the control group (*p =* 0.013) (Figure 6). The results of the death-censored graft survival subgroup analysis in the BMI groups were significant only for the DD group (*p* = 0.002) (Figure 7). Creatinine levels at 30 and 180 days post-transplant were worse for the obese patients (*p* < 0.001 and *p* = 0.031, respectively), but at the 1 and 3 year follow-up, there were no differences between the two groups. Rejection rates, both cellular and humoral, were not different between the two groups. On multivariate regression, IHD, recipient BMI, donor age, re-transplant, and graft type were independent risk factors for graft loss, whereas the risk factors for recipient death were recipient age, T2DM, IHD, and DD. Risk Factors of graft and patient survival are summarized in Table 3.

**Figure 8:**
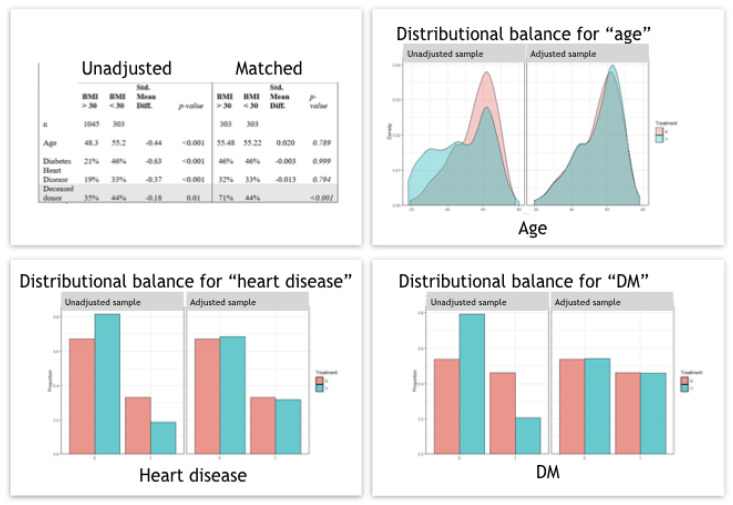
Propensity score matching analysis were included and adjusted for age, IHD, and T2DM.

**Figure 9:**
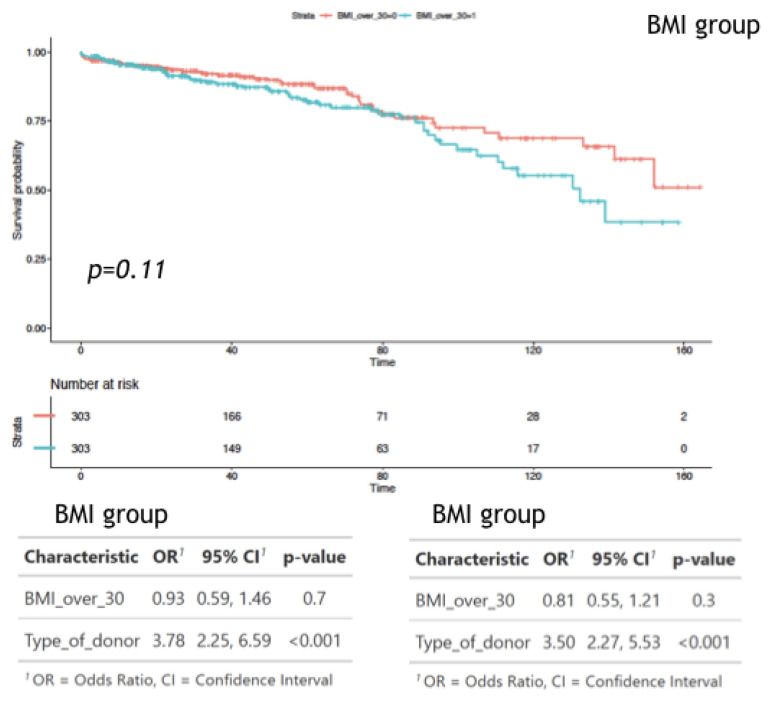
Propensity score matching analysis results, Kaplan–Meier Method with log rank test for comparison patient survival between groups.

In addition, a propensity score matching analysis was included and adjusted for age, IHD, and T2DM (Figure 8). The two matched cohorts included a total of 606 patients (303 each). Comparing outcomes between the two matched groups (BMI > 30 versus BMI < 30), further adjusting for donor type, resulted in an odds ratio (OR) of 0.81 for graft failure in the study group (95% confidence interval [CI] = 0.55,1.21; *p* = 0.3), and OR of 0.93 for death (95% CI = 0.59,1.46; *p* = 0.7), Figure 9.

## 4. Discussion

In this study, we compared the outcomes of kidney transplantation between patients with obesity (BMI > 30 kg/m^2^) and patients with a BMI ≤ 30 kg/m^2^, and further extended the comparison of the two groups between those who received LD and DD grafts. The results demonstrated that the risk for graft loss and death were significantly higher among the patients with obesity (*p* < 0.001). Similarly, the death-censored graft survival rates were lower in the patients with obesity and MO (*p* = 0.013). When analyzing the results according to graft type, LD vs. DD, a high BMI was also associated with significantly increased mortality (*p* < 0.001) in both subgroups of graft sources (LD, *p* = 0.045; DD, *p* = 0.004). However, when comparing graft survival between BMI groups in the LD and DD transplant, the difference was seen in the DD group (*p* = 0.002) but not in the LD group (*p* = 0.220).

Performing any major surgical procedure on obese patients is more difficult, takes longer, and is subject to a higher rate of operative and perioperative complications [13,14]. In addition, the outcomes of the same surgical procedures are worse for obese patients compared with their non-obese counterparts [2,15]. This is true also for patients undergoing transplant procedures. Meier-Kriesche et al. showed higher rates of mortality and graft failure in the long term in obese patients undergoing transplants [9]. On the contrary, in a meta-analysis published by Hill et al. [16], there was no significant difference in the risk of death between obese transplant recipients and those with a normal BMI. However, as indicated by the authors, there is a marginally greater risk of DGF and death-censored graft loss when comparing those two populations. Pascual et al. [17] observed that mortality among patients with a functioning allograft is usually related to cardiovascular comorbidities, which may have been present before transplantation [18]. For that reason, any analysis of transplant outcome should differentiate between graft loss due to progressive graft dysfunction from graft loss due to the death of a patient with a functioning allograft [19]. The finding of decreased death-censored graft survival in the patients with obesity and MO, and patients who received a DD graft, may be indicative of a negative impact of long-term dialysis on the results of a later transplant and the higher incidence of DGF among these patients, as compared to recipients of a LD graft. In a model for predicting DGF, the length of time the patient was on dialysis before transplant and the length of cold ischemic time for the graft were associated with an increased risk of graft failure [20]. Delayed graft function is known as a negative predictor of acute rejection and poorer long-term graft survival. It occurs in approximately 25% of DD kidney transplants and is associated with inferior graft survival [21]. In our cohort, DGF rates were higher in the study group with 30.7%, compared to controls of 17% (*p* < 0.001). The damage to the transplanted kidneys is assumed to be caused by multifactorial pathophysiological mechanisms, which are known to occur in obesity-related chronic kidney disease (CKD) in the native kidneys [22,23]. In a meta-analysis published by Hill et al., they concluded that the high risk for DGF in obese KT recipients has only a slightly increased risk for graft loss, compared with normal weight recipients [16]. However, in most of the studies, they could not include covariate’s analysis adjustments to type of donor and some of the recipient characteristics (such as age, gender, and comorbidity). That might impact both recipient and graft outcomes. Scheuermann et al. recently published that in the multivariate regression analysis, obesity (BMI ≥ 30) remained an independent predictor of DGF and postoperative surgical complications. However, when they adjusted for important covariates, obesity failed to be an independent predictor of decreased graft survival or acute rejection. They found that independent predictors of graft loss were recipient diabetes mellitus, hypertension, and kidneys from donors with expanded donor criteria [10]. The reported incidence of DGF is low in LD kidney transplants, reportedly ranging between 1% and 8% [24]. Our results show that graft and patient survival rates were significantly lower in patients with a BMI > 30 (*p* < 0.001) (Figure 2 and Figure 4). However, when comparing graft survival between BMI groups for LD and DD transplant, the difference was seen only in the DD group (*p* = 0.002). The patient survival in patients with a high BMI was associated with significantly increased mortality in both subgroups of graft sources (LD, *p* = 0.045; DD *p* = 0.004). However, in a prospective cohort study from French registries they presented a lower graft survival but similar patient survival in patients with grade 1 obesity [25].

Practice guidelines issued by the American Society of Transplantation [26] recommend a supervised weight loss regimen that includes a low-calorie diet, behavioral therapy, and a physical activity plan to achieve a target BMI of < 30 kg/m^2^ prior to a kidney transplantation. These guidelines also note that there are insufficient data to suggest which, if any, obese patients should be denied a transplant based on their obesity. Gill et al. [27] published a retrospective analysis of 702,456 incident ESRD patients aged 18–70 years (captured in the US Renal Data System between 1995 and 2007). The authors found that obesity impacted many inter-related considerations for transplant practice, including candidate selection, outcome prediction before and after transplant, and waitlist management. Consistent with those findings, a work by Segev et al. [28] reported that obese patients were less likely to receive a DD transplant after being listed and had a higher frequency of being placed on hold. Chan et al. [29] observed that morbidly obese patients with end-stage organ failure undergo more frustration and stress than non-obese patients before the transplant, and that may be delayed by the need to lose weight while waiting for a suitable organ.

Hoogeveen et al. reported that obesity (BMI > 30 kg/m^2^), both pre-transplantation and at 1-year post-transplantation, is a risk factor for both patient mortality and death-censored graft failure independent of other cardiovascular risk factors [11]. Our previous study [30] on 24 morbidly obese renal transplantation candidates who underwent bariatric surgery (BS) as a bridge to kidney transplantation revealed that BS can effectively and safely enable otherwise unsuitable patients to undergo kidney transplantation, with 16 of the 24 study subjects (67%) having proceeded to kidney transplantation following successful postoperative weight reduction. Those findings were supported by the results of several meta-analyses that demonstrated the superior efficacy of BS compared with non-surgical therapy in achieving sustained weight loss in morbidly obese patients in the general population [31,32].

The results of our study show inferior outcomes in patients with obesity/morbid obesity compared to recipients with normal weight and over-weight in DD transplants. Based on our experience, we suggest a strategy of addressing weight reduction intervention before the transplant when screening these candidates [30]. In our study, as in previous studies [10,33], we show that the risk factors for recipient death are recipient age, graft type (DD), and comorbidities such as T2DM and IHD. In addition, the risk factors for graft loss are IHD, recipient BMI, donor age, re-transplant, and graft type. This, again, shows that being overweight has a negative impact on graft survival in addition to factors associated with graft quality and immunological risks of re-transplantation. To better asses the effect of the various factors on the outcome we preformed propensity score matching analysis adjusted for age, IHD, and T2DM (Figure 8). It shows odds ratio (OR) of 0.81 for graft failure for a BMI > 30 (95% confidence interval [CI] = 0.55,1.21, *p* = 0.3), and OR of 0.93 for death (95% CI = 0.59,1.46, *p* = 0.7), Figure 9. The propensity score analysis shows also an OR of 3.78 for death among the high BMI group undergoing a DD transplant compared to LD transplantation (Figure 9). Thus, it seems that the main effect of obesity is among patients on the waiting list with other comorbidities and not in patients undergoing elective live-donor kidney transplantation who have a lower rate of cardiovascular risk and receive a higher quality graft.

Our study has several limitations that bear mention. First, it is retrospective in design and lacks anthropometric parameters for the entire cohort. Second, it has a relatively small number of morbidly obese patients. Third, it describes the experience of a single center. Fourth, we used BMI to define obesity, which may be an inappropriate measure to characterize the status of a patient. Therefore, further studies, with a measurement of body fat distribution and muscle mass, and their association with the risk of morbidity and mortality in transplant recipients, would be more appropriate [34]. Fifth, in the current study, we decided to exclude patients who underwent BS [30], but the influence of weight loss or gain on the KT outcome and possibilities of BS should be highlighted in future studies [35]. While the results are consistent with earlier studies, which showed that obesity is a risk factor for graft failure and death after kidney transplantation, this is one of the first investigations that compared the impact of DD and LD graft sources among different BMI groups and showed a negative association between recipient obesity and DD graft transplant in terms of graft survival. This negative impact of obesity was lessened among recipients of LD, which might be explained by their lower dialysis time prior to the transplant and having fewer cardiovascular comorbidities, and the fact that the LD graft began to function more rapidly than the DD graft in the majority of patients [24].

In summary, this longitudinal observational study with a mean follow-up of 55.5 months in a large cohort of kidney transplant recipients demonstrated that obesity is a risk factor for both mortality and graft loss. Deceased donor transplants have inferiority in this population in terms of graft survival. Based on propensity score matching analysis, recipient age and metabolic comorbidities associated with obesity are the main factors contributing to shorter patient survival time in obese populations. Our results emphasize the need for a finer evaluation when considering KT in patients with a BMI > 30 kg/m^2^, including comorbidities that are attributed to the increased risk. Type 2 diabetes mellitus and post-transplant diabetes mellitus should be carefully addressed in this population as a major factor of long-term results of transplant. Novel antidiabetic intervention among transplant patients, like GLP-1, seems to improve insulin requirements and weight but not survival outcomes, and should be further investigated [36,37,38]. Currently available bariatric procedures for weight loss prior or post kidney transplantation are recommended to achieve weight loss, leading to better long-term outcomes of obese patients [30,39,40].

## 5. Conclusions

Obesity of kidney graft recipients at the time of transplantation is a risk factor for graft loss and death in the long-term, specifically among patients who receive a deceased donor transplant after a long wait on dialysis with associated cardiovascular risks. Recipient age and metabolic comorbidities should be emphasized when evaluating patients with obesity as the main factors contributing to shorter patient survival time. Weight loss interventions using the new GLP-1 inhibitors and bariatric procedures in selected patients are advocated to prepare overweight patients for transplant.

## Figures and Tables

**Table 1:** Demographics and Characteristics of the Patients.

	Study Group Obese–Morbidly Obese	Control Group Normal Weight–Overweight	*p* Value
**Recipients, number**	314 (22.4%)	1089 (77.6%)	
**Age, (year) [mean ± SD]**	55.40 ± 11.94	48.43 ± 15.53	***p*** ** < 0.001**
**Gender, number**			*p* = 0.158
**Male**	224 (71.3%)	731 (67.1%)	
**Female**	90 (28.7%)	358 (32.9%)	
**BMI (kg/m^2^) ± SD**	33.25 ± 2.83	24.30 ± 3.18	***p*** ** < 0.001**
**Weight (kg) ± SD**	94.92 ± 12.37	69.97 ± 13.25	***p*** ** < 0.001**
**Comorbidity, number**			
**T2DM**	147 (46.8%)	235 (21.6%)	***p*** ** < 0.001**
**IHD**	100 (33%)	194 (18.5%)	***p*** ** < 0.001**
**Kidney disease, number**			***p*** ** < 0.001**
**T2DM**	101 (32.2%)	158 (14.5%)	
**HTN**	35 (11.1%)	56 (5.1%)	
**PKD**	30 (9.6%)	153 (14.0%)	
**FSGS**	29 (9.2%)	86 (7.9%)	
**GN**	21 (6.7%)	99 (9.1%)	
**Pyelonephritis**	5 (1.6%)	63 (5.8%)	
**Other**	29 (9.2%)	143 (13.1%)	
**Congenital**	4 (1.3%)	76 (7.0%)	
**Unknown**	45 (14.3%)	178 (16.3%)	
**Preemptive transplant**	49(17.7%)	265 (23.5%)	***p*** ** = 0.037**
**Re-transplant, number**	27 (8.6%)	188 (17.3%)	***p*** ** < 0.001**
**Donor age, mean ± SD**	47.67 ± 14.61	45.71 ± 13.95	***p*** ** = 0.005**
**Donor type, number**			***p*** ** = 0.008**
**Deceased**	137 (43.6%)	384 (35.2%)	
**Living**	177 (56.4%)	705 (64.7%)	
**Time on dialysis (months, mean ± SD)**	33.7 ± 39.6	37.0 ± 33.6	*p* = 0.180
**Time on dialysis, LD**	16.5 ± 24.2	19.6 ±24.0	*p* = 0.252
**Time on dialysis, DD**	60. 1 ± 29.9	65.2 ± 38.4	*p* = 0.158
**PRA class I (+), number (%)**	15 (5.5%)	75 (7.6%)	*p* = 0.103
**PRA class II (+), number (%)**	11(4%)	67 (6.8%)	*p* = 0.103
**HLA-A 0 match, number (%)**	142 (50.2%)	508 (49.4%)	*p* = 0.125
**HLA-B 0 match, number (%)**	164 (56.9%)	582 (56.5%)	***p*** ** = 0.016**
**HLA-DR 0 match, number (%)**	121 (45.7%)	409 (43.7%)	*p* = 0.575
**6 HLA mismatch, number (%)**	54 (17.2%)	212 (19.5%)	*p* = 0.901

BMI indicates body mass index; SD, standard deviation; T2DM, type 2 diabetes mellitus; IHD, ischemic heart disease; PKD, polycystic kidneys disease; FSGS, focal and segmental glomerulosclerosis; GN, Glomerulonephritis; PRA II and I, panel reactive antibody, class I and II; HLA-A/B/DR (0 match). Bold indicates significant.

**Table 2:** Kidney Transplant Outcomes.

	Study Group Obese-Morbidly Obese	Control Group Normal Weight- Overweight	*p* Value
**Recipients, number**	314 (22.4%)	1089 (77.6%)	
**LOS (days), mean ± SD**	14.28 ± 25.27	10.89 ± 12.849	***p*** ** = 0.002**
**DGF number**	95 (30.7%)	182 (17.0%)	***p*** ** < 0.001**
**PNF, number**	7 (2.3%)	8 (0.7%)	***p*** ** < 0.001**
**Rejection, number**			*p* = 0.197
**Cellular**	25 (8.0%)	64 (5.9%)	*p* = 0.228
**Humoral**	10 (3.2%)	19 (1.7%)	*p* = 0.175
Status of graft loss *	69 (22.3%)	148 (13.5%)	***p*** ** < 0.001**
Status death *	36 (11.7%)	84 (7.7%)	***p*** ** = 0.027**
**Creatinine (mg/dL) (total number of patients), mean ± SD**			
**30 days (n = 1318)**	1.78 ± 1.16	1.52 ±0.99	***p*** ** < 0.001**
**180 days (n = 1197)**	1.52 ± 0.89	1.41 ± 0.68	***p*** ** = 0.031**
**1 year (n = 1099)**	1.48 ± 0.80	1.38 ± 0.78	*p* = 0.097
**3 years (n = 656)**	1.79 ± 1.92	1.52 ± 1.40	*p* = 0.060

LOS indicates the length of stay; SD, standard deviation; DGF, delayed graft function; PNF, primary nonfunction. Bold indicates significant. * The outcome data of all recipients were censored on August 2019.

**Table 3:** Risk Factors for Graft Survival and Patient Survival after Kidney Transplantation.

	HR [Exp(B)]	95.0% CI for Exp(B) Lower	95.0% CI for Exp(B) Upper	*p* Value
**Cox regression for graft loss**				
**IHD (Y/N)**	1.892	1.496	2.687	***p*** ** < 0.001**
**BMI (kg/m^2^)**	1.038	1.010	1.066	***p*** ** = 0.007**
**Donor Age (year)**	1.018	1.008	1.028	***p*** ** < 0.001**
**1st/reTx (Y/N)**	1.750	1.177	2.602	***p*** ** = 0.006**
**LD/DD**	2.491	1.857	3.340	***p*** ** < 0.001**
**Cox regression for mortality**				
**Age (year)**	1.054	1.033	1.074	***p*** ** < 0.001**
**DM (Y/N)**	2.089	1.414	3.088	***p*** ** < 0.001**
**IHD (Y/N)**	1.761	1.192	2.600	***p*** ** = 0.004**
**LD/DD**	**2.682**	**1.801**	**3.992**	***p*** ** < 0.001**

IHD indicates ischemic heart disease; DM, diabetes mellitus; 1st/re-Tx, first/re-transplant; BMI, body mass index; kg, kilogram; yr, years; Y/N, yes/no; LD/DD, living donor/deceased donor. Bold indicates significant.

## Data Availability

The data that support the findings of this study are available from the corresponding author upon reasonable request.

## Abbreviations

BMI: body mass index; MO, morbid obesity; LD, living donor; DD, deceased donors; BS, bariatric surgery; ESRD, end-stage renal disease; PKD, polycystic kidneys disease; T2DM, type 2 diabetes; IHD, ischemic heart disease; PRA I/II, panel reactive antibody I and II; MM, HLA-A/B/DR/Total mismatch; rabbit antithymocyte globulin (ATG); hazard ratio (HR); odds ratio (OR); confidence interval (CI); chronic kidney disease (CKD); delayed graft function (DGF(; pri-mary non function (PNF) and kidney transplant (KT).
